# Life-Space Mobility in Heart Failure With Preserved Ejection Fraction

**DOI:** 10.1016/j.yjcafi.2025.12.013

**Published:** 2026-05-20

**Authors:** DYLAN MARSHALL, OMAR ZAINUL, MUSARRAT NAHID, KYLE LAPENNA, ASHKAN HASHEMI, PRINCESS OSMA, ALEXANDRA REICH, AIDAN SINHA, EMILY KIM, KATE ZARZUELA, PARAG GOYAL

**Affiliations:** 1 Department of Medicine, Columbia University Irving Medical Center, New York, New York; 2 Department of Medicine, Weill Cornell Medicine, New York, New York; 3 Program for the Care and Study of the Aging Heart, Weill Cornell Medicine, New York, New York.

**Keywords:** Heart failure with preserved ejection fraction, life-space, morbidity, mortality, prognosis

## Abstract

**Background:**

Life-space mobility quantifies an individual’s movement within their environment, ranging from one’s bedroom to outside of one’s geographic region. This makes life-space mobility an appealing yet understudied summary evaluation that can effectively integrate multiple health domains inclusive of conditions that are prevalent in heart failure with preserved ejection fraction (HFpEF). Whether life-space mobility is associated with adverse outcomes among patients with HFpEF who are ambulatory is unknown.

**Methods:**

We examined 175 consecutive patients with HFpEF seen at the Weill Cornell Medicine HFpEF program (November 2018 through March 2022). Life-space mobility was quantified via the Life-Space Assessment (LSA) and a simplified version of the LSA. The Kansas City Cardiomyopathy Questionnaire-12 and EuroQol 5-Dimension 5-level were used to examine the quality of life. The primary outcome was a 12-month composite of all-cause mortality and hospitalization. A Cox proportional-hazard model was used to examine the association between life-space mobility and the primary outcome, adjusting for race and the Meta-Analysis Global Group in Chronic Heart Failure score.

**Results:**

The median age was 76.8 years (interquartile range, 69.7–84.6 years). Most patients were female (67.4%) and of White race (65.1%). Those with low life-space mobility (based on both the LSA and simplified LSA) reported a worse quality of life (as measured by the Kansas City Cardiomyopathy Questionnaire-12 and EuroQol 5-Dimension 5-level). In addition, low life-space mobility (based on both the LSA and simplified LSA) was independently associated with all-cause hospitalization at 12-months in a fully adjusted model (LSA: hazard ratio, 2.41; 95% confidence interval, 1.23–4.69, *P* = .01; simplified LSA: hazard ratio, 2.46; 95% confidence interval, 1.19–4.31, *P* = .013).

**Conclusion:**

Among adults with HFpEF, life-space mobility tracks with multiple health deficits, parallels quality of life, and is associated with adverse clinical outcomes. The LSA and simplified LSA provide comparable prognostic information, suggesting that either could serve as a summary score of health and prognostic indicator among HFpEF.

## Lay Summary

Low life expectancy is linked with poor outcomes in adults with heart failure with preserved ejection fraction (HFpEF). Low life-space tracks with deficits spanning multiple health domains and quality of life. These findings were consistent whether life-space was assessed on the basis of the complete or simplified assessment tool. As such, the simplified Life-Space Assessment tool could serve as a brief, inexpensive, and efficient way to identify particularly vulnerable patients.

## Introduction

The unique vulnerabilities of older adults with heart failure with preserved ejection fraction (HFpEF) span multiple domains of health, prompting the recommendation for implementing a multidomain approach to optimally care for this population.^1,2^ More specifically, it has been recommended that the key health domains of medical, mind and emotion, physical function, and social environment, be assessed and incorporated into the care of older adults with HFpEF. Unfortunately, this approach is vastly underused, even among the strongest proponents of geriatric cardiology.^3^ Barriers to broad implementation include time constraints and lack of personnel to administer assessments, necessitating a novel approach to identifying and quantifying these deficits.

The notion of life-space mobility, introduced in 1985, quantifies community mobility ranging from one’s bedroom to outside of one’s geographic region.^4–6^ Previous data suggest that life-space mobility is impacted by multiple health domains,^7^ including comorbid conditions,^8–10^ mood and cognition,^11,12^ physical function,^13^ and social environment.^14,15^ This accordingly makes life-space mobility an appealing summary evaluation that can effectively integrate multiple health domains inclusive of conditions that are prevalent in HFpEF.^2,16^ Adding to its appropriateness as a potentially helpful clinical tool, life-space mobility is associated with a number of adverse outcomes important to community-dwelling older adults, including falls,^17^ loss of independence manifested by nursing home admissions,^5^ increased health care use,^18^ and mortality.^19^ To our knowledge, life-space mobility has not been studied in HFpEF. To better understand its prognostic role (and potential value in routine clinical care), we examined prospective data from a dedicated HFpEF Program in New York City to characterize life-space mobility and its association with adverse events in ambulatory adults with HFpEF.


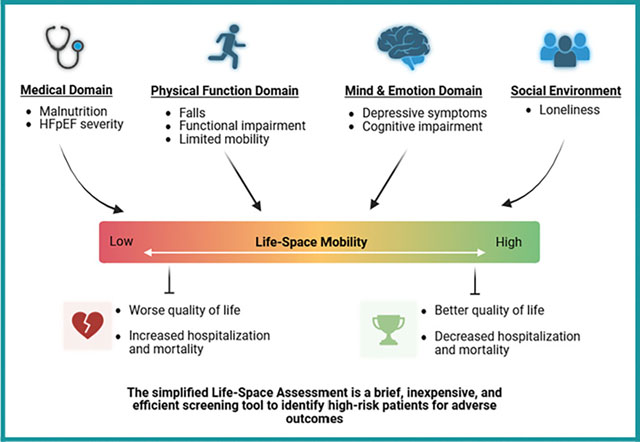


## Methods

### Study Population

The study population included consecutive patients with HFpEF who were ambulatory and had initial encounters at the Weill Cornell Medicine HFpEF program between November 2018 and March 2022. The patients cared for at this program include those with a previous diagnosis of HFpEF and those seeking evaluation for a potential new diagnosis of HFpEF. HFpEF was defined as (1) the presence of heart failure (HF) symptoms based on Framingham criteria^20^; (2) preserved left ventricular ejection fraction (LVEF) ≥50%^21^; and (3) the absence of alternative HF etiologies, including severe valvular disease, hypertrophic cardiomyopathy, pericardial disease, pulmonary arterial hypertension, cardiac amyloidosis, or other infiltrative diseases. Of note, the HFpEF Program did not evaluate any new patients between March and June of 2020 because of the COVID-19 pandemic.

### Multidomain Health Assessments

At the Weill Cornell Medicine HFpEF program, patients undergo multidomain health assessments,^22^ summarized in Supplemental Table S1, as guided by expert recommendations.^23^ The “legal sex” of the patient, noted in the electronic medical record, was used in this study. “Legal sex” is what an individual uses for their sex on official documents like a passport. This may differ from “sex assigned at birth”. These data are inputted into a registry that has been approved by the Weill Cornell Medicine Institutional Review Board.

For the medical domain, HF severity was determined using the validated Meta-Analysis Global Group in Chronic Heart Failure (MAGGIC) mortality risk score, with greater scores indicating greater risk for poor outcomes. Malnutrition was determined using the validated Mini-Nutritional Assessment Short Form (MNA-SF)^24^ and defined as MNA-SF scores ≤11.^25^ Polypharmacy was defined as taking ≥10 medications, which has been associated with adverse events in multiple HF studies.^26,27^ Hearing and vision impairment were assessed via self-report—patients with moderate to worse hearing and/or vision impairment were deemed as having a sensory impairment.

For the physical function domain, limited mobility was assessed using the 5-meter gait speed test. Patients with gait speed ≥6 seconds were classified as having limited mobility, as this threshold has been validated as an independent predictor of morbidity and mortality among patients with cardiovascular disease^28,29^ including HFpEF.^30^ Falls were assessed by self-report of a fall within the past year. Lastly, functional impairment was determined using the validated Katz Activities of Daily Living assessment.^31^ Patients with a score of 0–2 were classified as having severe functional impairment, scores 3–5 were classified as having moderate functional impairment, and a score of 6 was classified as having no functional impairment.

For the mind and emotion domain, cognitive impairment was assessed using the Mini-Cog. Mini-cog scores ≤2 were used to define patients with cognitive impairment.^32^ Depressive symptoms were assessed on the basis of the Patient Health Questionnaire 8 (PHQ-8), which is a validated screening tool for depressive symptoms. The presence of depressive symptoms was defined by PHQ-8 scores ≥5, a threshold that is associated with adverse outcomes in HFpEF.^16^

For the social environment domain, loneliness was assessed using the UCLA 3-item loneliness survey. This survey consists of 3 questions that assess 3 dimensions of loneliness (relational connectedness, social connectedness, and self-perceived isolation), and is scored on a scale from 3 to 9, with scores ≥6 indicating loneliness.^33^

### Exposure: Life-Space Mobility

The primary exposure of interest was life-space mobility. Data on life-space mobility are routinely collected by administering the Life-Space Assessment (LSA) to all new patients seen at the Weill Cornell Medicine HFpEF program. These data are then inputted into an institutional review board–approved registry.

The LSA was created in 2003 and assesses community mobility by quantifying the frequency of movement between life-space levels using standardized definitions and the degree of assistance needed to reach those levels in the previous 4 weeks.^34^ The LSA specifically asks: “During the past 4 weeks, have you: been to other rooms in your home besides the room where you sleep (Level 1); been to an area outside your home such as your porch, deck, or patio, the hallway of an apartment building, or garage (Level 2); been to places in your neighborhood other than your own yard or apartment building (Level 3); been to places outside your neighborhood but within your town (Level 4); and been to places outside your town? (Level 5).” For every life-space level, patients are asked how many days during the week they reached that location and whether they needed help from an assistive device or another person.

For this study, we quantified life-space mobility in 2 different ways. First, we reported life-space mobility based on the traditional scoring of the LSA, which is a composite score that is calculated on the basis of life-space level, frequency of attaining each level, and degree of independence in achieving each level. LSA composite scores range from 0 to 120, with greater scores representing greater community mobility. For the purposes of these analyses, we examined tertiles of LSA scores.

Second, we examined life-space mobility using a simplified LSA modeled according to Zammit et al.^35^ The score for the simplified LSA scale is calculated on the basis of whether patients have been to a particular level within the environment in the past 4 weeks (Supplemental Material). The levels are defined as level 1, movement to rooms outside of one’s bedroom; level 2, movement to an area outside of your home such as a porch, deck, or driveway; level 3, movement to places outside of the home in one’s neighborhood; level 4, movement to locations outside of the neighborhood in one’s town; and level 5, movement to locations outside of one’s hometown. The simplified LSA score for each patient is the count of affirmative responses to each level, generating a cumulative score that ranges from 0 (not leaving the bedroom where one sleeps) to 5 (outside of town). For the purposes of these analyses, we divided patients into 3 groups: small life-space (limited to level 2 or less), medium life-space (limited to level 4 or less), and large life-space (level 5).

### Primary Outcome

The primary outcome was a composite of all-cause mortality and all-cause nonelective hospitalization over a 1-year follow-up period after baseline administration of the LSA. Outcomes were ascertained from the electronic medical record. Because the electronic medical record incorporates nearly all encounters across the health care enterprise and integrates an extensive network of patient encounters across hospital systems in the tristate area via a shared electronic medical record system, loss to follow-up was rare. Moreover, most patients included in this study were seen numerous times within the HFpEF Program throughout this study, providing ample encounters within the electronic medical record to ascertain outcomes.

### Statistical Analysis

Continuous variables are expressed as median and interquartile range (IQR): differences were compared using the Kruskal-Wallis test. Categorical variables were expressed as count and percentage and assessed using the Pearson χ^2^ or Fisher exact test. We created a radar plot to visualize the relationship between life-space mobility and the following health measures: HFpEF severity (based on the MAGGIC risk prognostic score, which ranges from 0 to 57, where greater numbers indicate worse prognosis), malnutrition (based on the MNA-SF), sensory impairment defined as hearing and/or vision impairment (based on self-report on a 6-item Likert scale), cognitive impairment (based on the Mini-Cog), depressive symptoms (based on the PHQ-8), limited mobility (based on the 5-meter gait speed test), falls within the previous year, functional impairment (based on Katz Index of Independence in Activities of Daily Living scores), and loneliness (based on the UCLA 3-item loneliness scale).

We created box plots and scatterplots to examine the relationship between life-space mobility and quality of life using the 12-item Kansas City Cardiomyopathy Questionnaire (KCCQ-12) and the European Quality of Life 5 Dimensions 5 Level Version. For box plots, the center line in each box represents the 50th percentile (median); the bottom of each box represents the 25th percentile; the top of each box represents the 75th percentile; the bottom “whisker” is equal to the 25th percentile minus 1.5 times the interquartile range; and the upper “whisker” is equal to the 75th percentile plus 1.5 times the interquartile range.

Kaplan-Meier curves were used to examine the composite outcome of 1-year all-cause hospitalization and all-cause mortality according to life-space mobility. To evaluate associations of life-space mobility with the composite outcome, we conducted Cox proportional hazard model analysis adjusting for race and severity of illness using the MAGGIC prognostic risk score. The MAGGIC prognostic risk score has been validated in HFpEF as a predictor of morbidity and mortality^36^ and includes the following variables: age, sex, (defined as “male or female”) diabetes mellitus, chronic obstructive pulmonary disease, smoking status, body mass index, systolic blood pressure, creatinine, LVEF, first HF diagnosis within previous 18 months of baseline visit, New York Heart Association class, and current beta-blocker, angiotensin-converting enzyme inhibitor, or angiotensin receptor blocker usage. Because race is not included in the MAGGIC score and may be an important confounder, we adjusted for White vs non-White race. To evaluate the linearity of the relationship (and determine the potential need for restricted cubic splines for nonlinear curves), we used the likelihood ratio (LR) test between the spline model and the linear model (with the continuous exposure but without spline terms), where a significant LR test indicates a nonlinear relationship. A *P* value of < .05 was considered statistically significant for all analyses. Statistical analyses were performed with R 4.2.2 (R Foundation for Statistical Computing, Vienna, Austria). Dr Goyal had full access to all the data in this study and takes full responsibility for the integrity of the data and the accuracy of the data analysis.

## Results

Among 175 eligible patients, the median age was 76.8 years (IQR, 69.7–84.6), and the majority were female (67.4%) and White (65.1%). The most common comorbid conditions were hypertension (80.0%), atrial fibrillation (45.7%), diabetes (40.6%) and coronary artery disease (30.9%). The median LVEF was 63.0% (IQR, 59.0–69.0%) and the median MAGGIC score was 24.0 (IQR, 19.0–28.0). During the 1-year follow-up, 68 (38.9%) patients experienced the primary outcome—of note, all 68 primary outcome events were a hospitalization. The distribution of outcomes by year is shown in Supplemental Table S2.

### Life-Space Assessment

The median LSA scores were 19 in the low LSA tertile, 44 in the middle LSA tertile, and 80 in the high LSA tertile. Compared with patients in the high LSA tertile, those in the low LSA tertile were older, more likely to be female, more likely to have chronic obstructive pulmonary disease, and were more likely to have more advanced HF on the basis of New York Heart Association class and MAGGIC scores (Table 1). The distribution of scores on the basis of study year is shown in Supplemental Table S3. Of note, among the 12 patients who were excluded from this analysis because of missing data on follow-up, 7 were in the low LSA tertile, 1 was in the middle LSA tertile, and 4 were in the high LSA tertile.

**Fig. 1.**
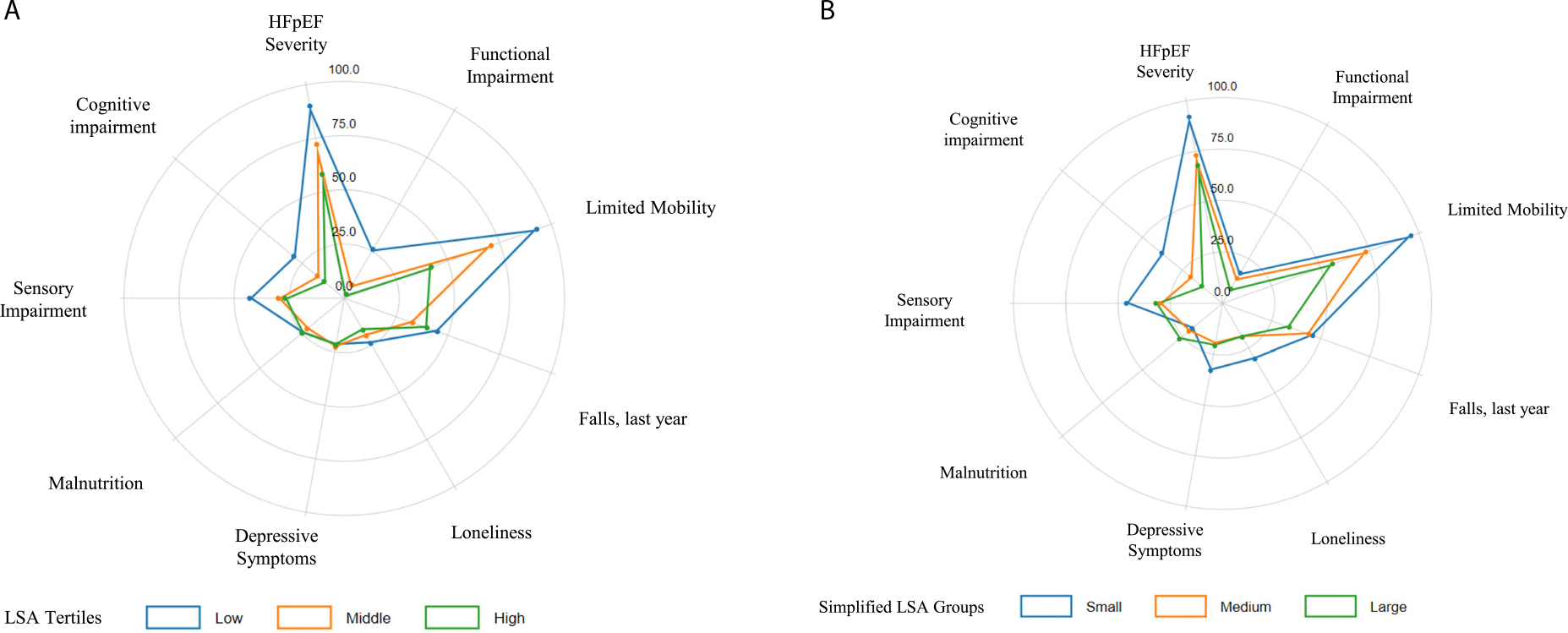
Radar plots showing the percentage of patients with health deficits according to LSA tertiles (A) and simplified LSA groups (B). HFpEF, heart failure with preserved ejection fraction; LSA, Life-Space Assessment.

Regarding health domains, patients in the low LSA tertile more frequently had polypharmacy, hearing and vision impairment, cognitive impairment, limited mobility, functional impairment, falls, and loneliness compared with those in the high LSA tertile (Table 2). Figure 1A shows that patients in the low LSA tertile experienced greater deficits across the health domains compared with patients in the high LSA tertile.

**Fig. 2.**
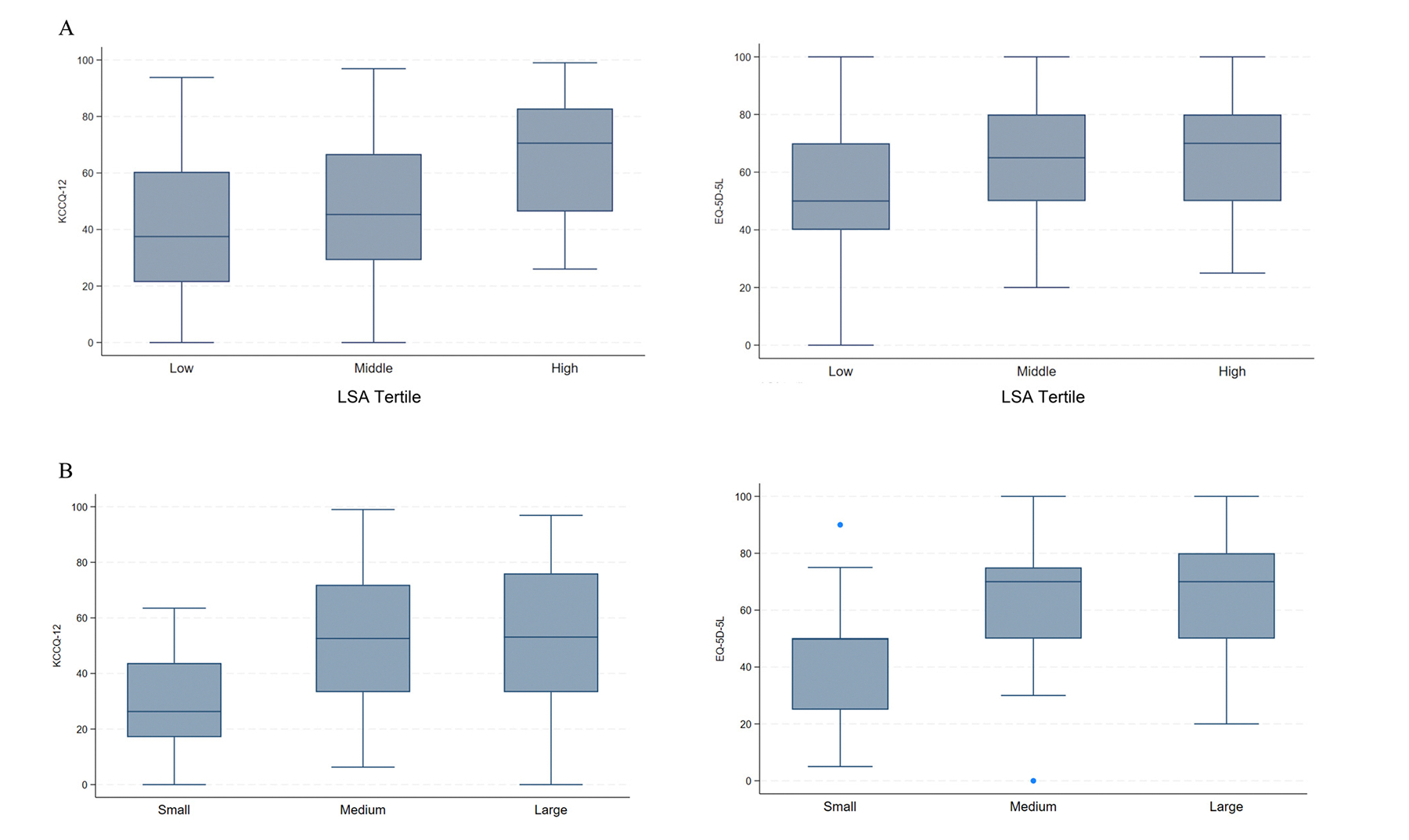
Box plots displaying the KCCQ-12 and EQ-5D-5L scores (mean, 95% confidence intervals [CIs]) across levels of LSA tertiles (A) and simplified LSA groups (B). KCCQ-12 and EQ-5D-5L scores (mean, 95% CIs) across levels of simplified LSA groups EQ5D5L, European Quality of Life 5 Dimensions 5 Level; KCCQ-12, 12-item Kansas City cardiomyopathy questionnaire; LSA, Life-Space Assessment.

Patients in the low LSA tertile had a median KCCQ-12 score of 37.5 (IQR, 21.4–60.4), which was significantly lower than patients in the high LSA tertile who had a median score of 70.6 (IQR, 46.3–82.8), *P* < .001. Patients in the low LSA tertile also had a median EQ-5D-5L score of 50.0 (IQR, 40.0–70.0), which was significantly lower than patients in the high LSA tertile, who had a median score of 70.0 (IQR, 50.0–80.0, *P* < .001; Fig. 2A). Scatter plots are shown in Supplemental Figures S1–2

**Fig. 3.**
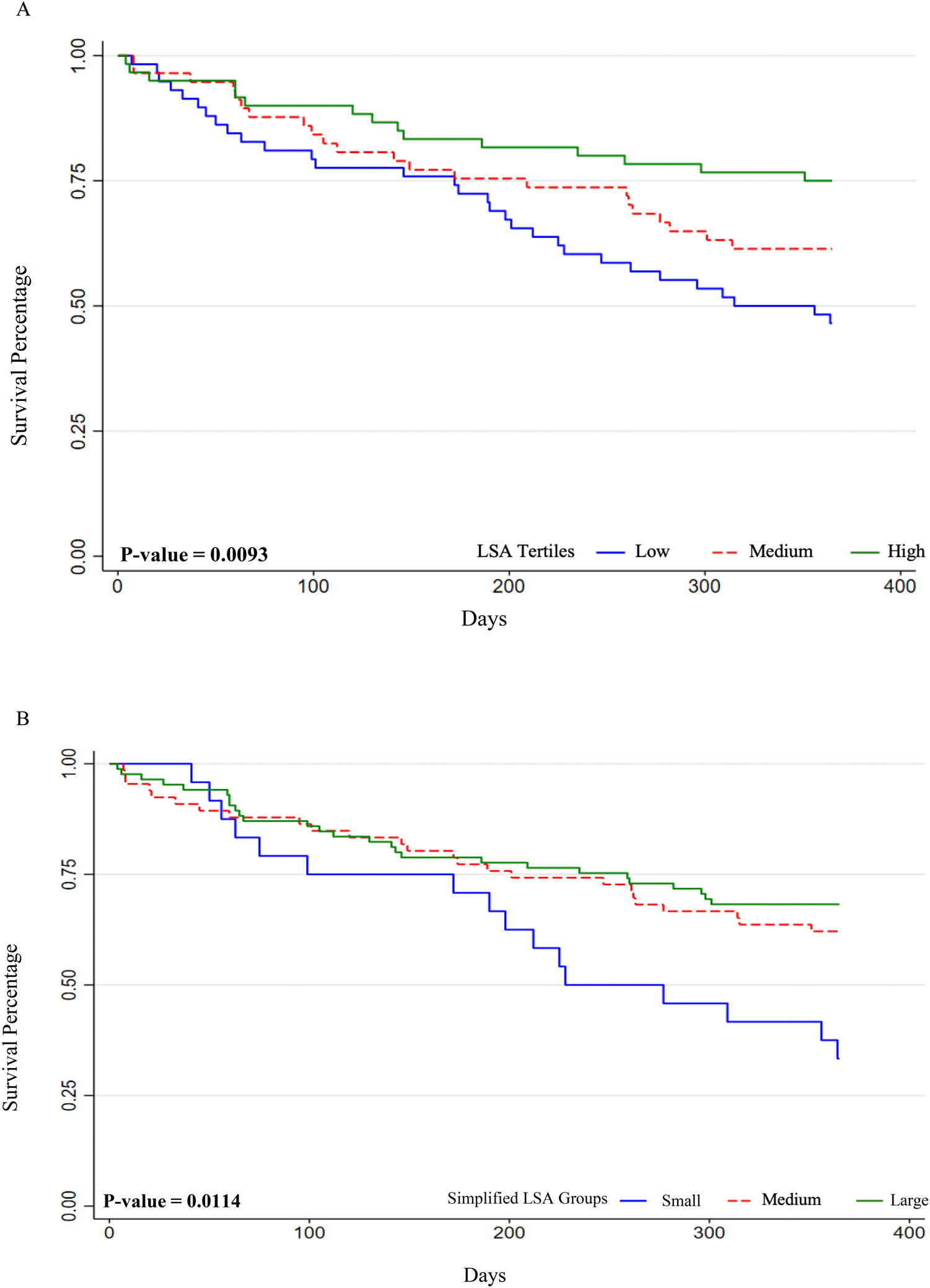
Kaplan–Meier curve for the composite outcome of 1-year all-cause hospitalization and all-cause mortality according to LSA tertiles (A) and simplified LSA groups (B). LSA, Life-Space Assessment.

As shown in Figure 3A, patients in the low LSA tertile experienced more all-cause hospitalizations than those in the high LSA tertile, (unadjusted hazard ratio [HR], 2.53; 95% confidence interval [CI], 1.36–4.68, P = .0093). In an adjusted model, patients in the low LSA tertile had greater than a 2-fold increased hazard for all-cause hospitalization compared with patients in the high LSA tertile (adjusted HR, 2.41; 95% CI, 1.23–4.69, P = .01).

### Simplified LSA

On the basis of the simplified LSA, 13.7% of patients had a small life-space, 37.7% had a medium life-space, and 48.6% had a large life-space. Compared with patients with large life-space, those with small life-space were more likely to have diabetes and greater MAGGIC scores (Table 1). The distribution of scores based on study year is shown in Supplemental Table S3. Of note, among the 12 patients who were excluded from this analysis because of missing data on follow-up, 4 had small life-spaces, 2 had medium life-spaces, and 6 had high life-space.

Regarding health domains, patients with small life-space more frequently had cognitive impairment and limited mobility compared with those with large life-space (Table 2). Figure 1B shows that patients with small life-space experienced greater deficits across the health domains compared with patients with large life-space.

Patients with small life-space had median KCCQ-12 scores of 26.3 (IQR, 17.1– 43.8), which was significantly lower than patients with large life-space who had a median score of 53.1 (IQR, 33.3–76.0, *P* < .001). Patients with small life-space had a median EQ-5D-5L score of 50.0 (IQR, 25.0–50.0), which was also significantly lower than patients with large life-space who had a median score of 70.0 (IQR, 50.0–80.0, *P* < .001) (Fig. 2B). Box plots for each individual simplified LSA score are shown in Supplemental Figures S3–4.

As shown in Figure 3B, patients with small life-space experienced more all-cause hospitalization than those with large life-space, (unadjusted HR, 2.47; 95% CI, 1.33–4.59, *P* = .004). In an adjusted model, patients with small life-space had greater than a 2-fold increased hazard for all-cause hospitalization compared with large life-space, (adjusted HR, 2.46; 95% CI, 1.19–4.31, *P* = .01).

A plot of HRs for the primary outcome according to LSA and simplified LSA as continuous variables is shown in Supplemental Figures S5–6. Of note, the LR tests for nonlinearity were not statistically significant (LSA model: *P* = .14; simplified LSA model: *P* = .22), indicating that the relationship between the exposure and the outcome is not better captured by nonlinear functions (such as restricted cubic splines).

## Discussion

In this study of ambulatory patients with HFpEF, we found that life-space mobility was associated with all-cause hospitalization, even after adjusting for race and the MAGGIC prognostic risk score. We also found that life-space mobility mirrors deficits across multiple health domains (especially physical function) and impairments in quality of life. Notably, we found these relationships whether life-space was measured by the LSA or a simplified version of the LSA.

Since its inception in 1985, the importance of life-space mobility as a concept has been gaining appreciation in the geriatric literature.^7,37,38^ Life-space mobility is influenced by factors both within and outside the control of an individual: medical, mind and brain, physical function, and social environmental factors all impact the way individuals are able to carry out their lives.^7^ In theory, life-space mobility represents the nexus of multiple health domains among older adults. Our findings here confirm this by showing that life-space mobility tracks with deficits across multiple health deficits, provides important prognostic information, and parallels quality of life. Importantly, these findings indicate that life space can effectively represent a summary of multiple health deficits among patients with HFpEF. This raises the notion that life-space may be particularly well-suited as a routine tool for use among patients with HFpEF. It is well-known that patients with HFpEF experience deficits that span multiple health domains^1,2^ and yet, these deficits may not be well-characterized, given the challenges of implementing multi-domain assessments.^3^ On the basis of data here, it may be reasonable to use the life-space as a summary tool of health, and perhaps even a screening tool which can prompt a more detailed evaluation of individual health domain and subdomains when low.

The first tool developed to quantify the life-space mobility idea was the Life Space Questionnaire. First developed in 1999, the Life Space Questionnaire assesses an individual’s movement across predetermined zones in the prior 3-day period. Then in 2003, a more detailed tool called the LSA was developed, which incorporated movement across predetermined zones and added frequency of this movement and whether this movement required assistance. Ultimately, the LSA yields more granular data and is the most used life-space mobility assessment tool in the geriatric literature. Moreover, the LSA has been translated and validated for use in 8 languages,^7^ and normative values have been published for comparison of cohorts in the United States.^4^ Limited life-space mobility based on the LSA has shown associations with a range of medical conditions including cognitive decline,^39^ falls,^40^ health care use,^41^ and mortality.^42^ The LSA has also been used to demonstrate the association between limited life-space and poor outcomes in patients with other common medical problems like peripheral arterial disease,^8^ chronic kidney disease,^9^ and chronic obstructive pulmonary disease.^10^ Despite this, one of the important drawbacks of the LSA is that it takes almost 10 minutes to complete. This poses a practical concern regarding the routine use of the LSA in clinical practice.^43^ Given its relevance as a summary metric for health, a simplified version may be useful. We accordingly examined whether a simplified LSA would be sufficient in predicting outcomes, and showed that its association with adverse outcomes (and reflection of health deficits and quality of life) was similar to the more extensive LSA tool among ambulatory adults with HFpEF. This suggests that the simplified LSA could serve as a short, inexpensive, and efficient way to identify significantly impaired patients who have multiple health deficits and are at high risk for poor outcomes.^44^ Future work to incorporate the simplified LSA (perhaps supplemented by wearables), identify optimal thresholds, and determine whether its use can benefit clinicians and patients with HFpEF are thus warranted.

### Limitations

There were a few limitations to our study. Our results are from a single-center specialty program at a quaternary academic center, which may limit generalizability. Our study was limited by a small sample size (n = 175); larger studies that capture the national and international HFpEF populations are needed to validate these findings. Also, our findings are vulnerable to recall bias as well as the inherent limitation of chart review. This includes the possibility of inaccurate or missing data related to outcomes.

## Conclusion

Our study found that restricted life-space mobility is common among older adults with HFpEF; and that life-space mobility tracks with multiple health deficits, parallels quality of life, and is associated with all-cause hospitalization. These findings suggest that life-space mobility could serve as an important summary measure for health that may be beneficial to incorporate into the routine clinical care of patients with HFpEF.

## Supplementary Material

1

2

Supplementary material associated with this article can be found in the online version at doi:10.1016/j.yjcafi.2025.12.013.

## Figures and Tables

**Table 1:** Baseline demographics stratified by Life-Space Assessment (LSA) tertiles and simplified LSA groups

Characteristic, n (%)	Overall, N = 175	LSA Tertiles			*P* Value*	Simplified LSA Groups			*P* Value*
		Low (n = 58)	Medium (n = 57)	High (n = 60)		Small (n = 24)	Medium (n = 66)	Large, (n = 85)	

Sociodemographics									
Age, y, median (IQR)	76.8 (69.7-84.6)	78.9 (73.2-87.5)	77.4 (68.6-84.6)	74.1 (68.6-81.2)	.035	77.6 (72.8-82.6)	78.2 (69.7-86.9)	76.0 (69.2-84.2)	.44
Female legal sex	118 (67.4%)	43 (74.0%)	43 (75.0%)	32 (53.0%)	.016	16 (67.0%)	50 (76.0%)	52 (61.0%)	.16
Primary race					.43				.31
White	114 (65.1%)	35 (60.0%)	35 (61.0%)	44 (73.0%)		13 (54.0%)	41 (62.0%)	60 (71.0%)	
African American	29 (16.6%)	12 (21.0%)	11 (19.0%)	6 (10.0%)		7 (29.0%)	11 (17.0%)	11 (13.0%)	
Asian	7 (4.0%)	4 (7.0%)	1 (2.0%)	2 (3.0%)		2 (8.0%)	4 (6.0%)	1 (1.0%)	
Other	23 (13.1%)	6 (10.0%)	10 (18.0%)	7 (12.0%)		2 (8.0%)	9 (14.0%)	12 (14.0%)	
Unknown	2 (1.1%)	1 (2.0%)	0 (0.0%)	1 (2.0%)		0 (0.0%)	1 (2.0%)	1 (1.0%)	
Hispanic ethnicity	19 (10.9%)	8 (14.0%)	7 (12.0%)	4 (7.0%)	.42	3 (12.0%)	7 (11.0%)	9 (11.0%)	.96
Comorbid conditions									
Coronary artery disease	54 (30.9%)	18 (31%)	19 (33%)	17 (28%)	.84	9 (38.0%)	20 (30.0%)	25 (29.0%)	.74
Atrial fibrillation	80 (45.7%)	26 (45.0%)	27 (47.0%)	27 (45.0%)	.95	12 (50.0%)	34 (52.0%)	34 (40.0%)	.33
Diabetes mellitus	71 (40.6%)	31 (53.0%)	19 (33.0%)	21 (35.0%)	.050	17 (71.0%)	23 (35.0%)	31 (36.0%)	.005
Hypertension	140 (80.0%)	48 (83.0%)	45 (79.0%)	47 (78.0%)	.81	20 (83.0%)	54 (82.0%)	66 (78.0%)	.74
COPD	38 (21.7%)	19 (33.0%)	14 (25.0%)	5 (8.0%)	.005	9 (38.0%)	15 (23.0%)	14 (16.0%)	.09
BMI, kg/m^2^, median (IQR)	30.6 (26.2-36.9)	30.8 (26.2-37.7)	31.3 (27.8-36.7)	28.7 (25.3-35.0)	.30	31.5 (26.0-38.9)	30.2 (25.9-37.7)	30.8 (26.4-36.6)	.81
Systolic BP, mm Hg, median (IQR)	132.0 (120.0-146.0)	134.5 (121.0-161.0)	130.0 (120.0-142.0)	133.5 (120.0-145.0)	.28	140.0 (120.5-164.5)	131.0 (120.0-144.0)	133.0 (122.0-145.0)	.40
HF characteristics									
HF diagnosed in the previous 18 mo	112 (64.0%)	33 (57.0%)	39 (68.0%)	40 (67.0%)	.38	15 (62.0%)	37 (56.0%)	60 (71.0%)	.18
NYHA class					.001				.32
I	7 (4.0%)	0 (0.0%)	2 (4.0%)	5 (8.0%)		0 (0.0%)	1 (2.0%)	6 (7.0%)	
II	68 (38.9%)	16 (28.0%)	19 (33.0%)	33 (55.0%)		7 (29.0%)	25 (38.0%)	36 (42.0%)	
III	95 (54.3%)	39 (67.0%)	35 (61.0%)	21 (35.0%)		16 (67.0%)	39 (59.0%)	40 (47.0%)	
IV	5 (2.9%)	3 (5.0%)	1 (2.0%)	1 (2.0%)		1 (4.0%)	1 (2.0%)	3 (4.0%)	
LVEF, median (IQR)	63.0 (59.0-69.0)	65.0 (60.0-70.0)	63.0 (58.0-67.0)	62.0 (58.0-69.5)	.21	63.5 (60.0- 67.5)	65.0 (60.0-70.0)	60.0 (57.0- 67.0)	.004
MAGGIC score, median (IQR)	24.0 (19.0-28.0)	26.0 (23.0-30.0)	23.0 (19.0-27.0)	21.0 (16.0-26.0)	<.001	26.5 (23.5- 29.5)	24.0 (19.0-28.0)	22.0 (19.0-27.0)	.04
KCCQ score, median (IQR)	49.5 (29.2-74.0)	37.5 (21.4-60.4)	45.3 (29.2- 66.7)	70.6 (46.3-82.8)	<.001	26.3 (17.1-43.8)	52.6 (33.3-71.9)	53.1 (33.3-76.0)	<.001
EQ-5D-5L score, Median (IQR)	65.0 (50.0-75.0)	50.0 (40.0-70.0)	65.0 (50.0- 80.0)	70.0 (50.0-80.0)	<.001	50.0 (25.0-50.0)	70.0 (50.0-75.0)	70.0 (50.0-80.0)	<.001
1-y composite of all-cause hospitalization or mortality	68 (38.9%)	31 (53.0%)	22 (39.0%)	15 (25.0%)	.007	16 (67.0%)	25 (38.0%)	27 (32.0%)	.008

Values are median (IQR); n (%).

ADL, activities of daily living; BMI, body mass index; BP, blood pressure; COPD, chronic obstructive pulmonary disease; EQ-5D-5L, EuroQol 5-Dimension, 5-Level; HF, heart failure; KCCQ-12, Kansas City Cardiomyopathy Questionnaire-12; LVEF, left ventricular ejection fraction; MAGGIC, Meta-Analysis Global Group in Chronic Heart Failure; MNA-SF, Mini Nutritional Assessment-Short Form; NYHA, New York Heart Association; PHQ-8, Patient Health Questionnaire-8; UCLA, University of California, Los Angeles.

* Kruskal-Wallis test; Pearson χ^2^ test; Fisher exact test.

**Table 2:** Prevalence of affected health domains stratified by Life-Space Assessment (LSA) tertiles and simplified LSA groups

Characteristic, n (%)	Overall, N = 175	LSA Tertiles			*P* Value*	Simplified LSA Groups			*P* Value*
		Low, n = 58	Middle, n = 57	High, n = 60		Small, n = 24	Medium, n = 66	Large, n = 85	

Medical domain									
Malnutrition	35 (24%)	11 (25%)	11 (22%)	13(25%)	.88	3(19%)	12(21%)	20 (27%)	.66
Sensory Impairment	58 (33%)	25 (43%)	17 (30%)	16 (27%)	.13	11 (46%)	20 (30%)	27 (32%)	.36
Hearing loss	17 (10%)	11 (19%)	3 (5%)	3 (5%)	.014	4 (17%)	6 (9%)	7 (8%)	.46
Vision loss	47 (27%)	17 (29%)	15 (26%)	15 (25%)	.86	7 (29%)	16 (24%)	24 (28%)	.83
Polypharmacy	80 (50%)	30 (57%)	29 (57%)	21 (38%)	.084	13 (59%)	34 (58%)	33 (42%)	.14
Mind and emotion domain									
Cognitive impairment	33 (19%)	17 (30%)	9 (16%)	7 (12%)	.033	9 (38%)	13 (20%)	11 (13%)	.03
Depressive symptoms	39 (22%)	13 (22%)	13 (23%)	13 (22%)	.99	8 (33%)	13 (20%)	18 (21%)	.37
Physical function domain									
Limited mobility	115 (68%)	51 (93%)	39 (71%)	25 (42%)	<.001	22 (96%)	45 (73%)	48 (56%)	.001
Functional impairment					<.001				.27
No impairment (6)	147 (85%)	39 (68%)	50 (88%)	58 (97%)		17 (74%)	54 (82%)	76 (89%)	
Moderate impairment (3—5)	15 (9%)	9 (16%)	5 (9%)	1 (2%)		4 (17%)	6 (9%)	5 (6%)	
Severe impairment (0—2)	12 (7%)	9 (16%)	2 (4%)	1 (2%)		2 (9%)	6 (9%)	4 (5%)	
Fall, last year	69 (39%)	26 (45%)	19 (33%)	24 (40%)	.45	11 (46%)	29 (44%)	29 (34%)	.37
Social environment domain									
Loneliness	28 (20%)	10 (24%)	10 (20%)	8 (17%)	.73	5 (31%)	10(19%)	13 (19%)	.50

Abbreviations as in Table 1.

* Pearson χ^2^ test; Fisher exact test.
