# Supplementary material for: Life-Space Mobility in Heart Failure With Preserved Ejection Fraction
Source: J Card Fail Intersect. Author manuscript; Available in PMC 2026 Jul 29. (PMC13410940; doi:10.1016/j.yjcafi.2025.12.013)
Supplement: 1 [file NIHMS2179308-supplement-1.docx]

Life-Space Mobility Supplemental File

Supplemental Table 1: Summary of Multi-Domain Health Assessments Performed at the WCM HFpEF Program

| **Health Domain** | **Variable** | **Measurement Tool** | **Cutoff / Definition** |
| --- | --- | --- | --- |
| Medical | Malnutrition | Mini-Nutritional Assessment Short Form (MNA-SF) | MNA-SF ≤ 11 |
|  | Sensory Impairment | Self-reported hearing/vision loss | Moderate or worse impairment |
|  | Polypharmacy | Medication Count | ≥ 10 medications |
|  | Cognitive Impairment | Mini-Cog Test | Mini-Cog ≤ 2 |
| Mind & Emotion | Depressive Symptoms | Patient Health Questionnaire-8 (PHQ-8) | PHQ-8 ≥ 5 |
|  |  |  |  |
|  | Limited Mobility | 5-Meter Gait Speed Test | Gait speed ≥ 6 sec |
| Physical Function | Functional Impairment | Katz Activities of Daily Living (ADL) | Severe: ADL score 0-2 \| Moderate: ADL score 3-5 \|  No Impairment: ADL score 6 |
|  | Falls | Self-report | Any fall in past year |
| Social Environment | Loneliness | UCLA 3-Item Loneliness Survey | Score ≥ 6 |

| **Study Year** | **Outcome** |
| --- | --- |
| 2018 | 2 (2.9%) |
| 2019 | 17 (25%) |
| 2020 | 16 (23.5%) |
| 2021 | 13 (19.1%) |
| 2022 | 15 (22.1%) |
| 2023 | 5 (7.4%) |
|  |  |

Supplemental Table 2: Distribution of the Primary Outcome according to Study Year

Supplemental Table 3: Median LSA and Simplified LSA according to Study Year

| **Study Year** | **LSA, Median (IQR)** | **Simplified LSA, Median (IQR)** |
| --- | --- | --- |
| 2018 | 46 (20-60) | 4 (2-4) |
| 2019 | 44 (23-75) | 5 (3-5) |
| 2020 | 48 (30.5-64.5) | 5 (3-5) |
| 2021 | 42 (22.5-64) | 4 (3-5) |
| 2022 | 36.5 (24-69) | 4 (3-5) |
|  |  |  |

Supplemental Material: A Summary of the Simplified Life-Space Assessment Conducted on Patients seen in the WCM HFpEF Clinic

**Question: In the past 4 weeks, have you been to…**


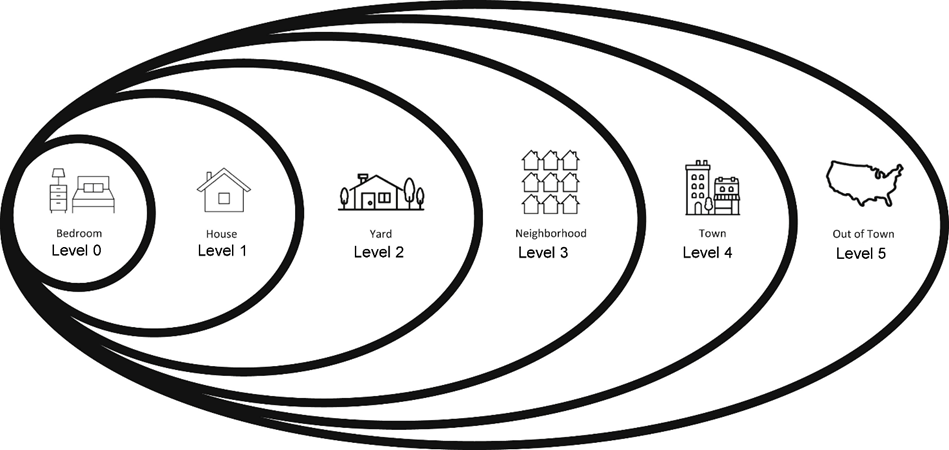


| **Location** | **Yes** | **No** |
| --- | --- | --- |
| **Level 0** — Bedroom only (did not leave bedroom) | ☐ | ☐ |
| **Level 1** — Rooms outside your bedroom (elsewhere in your home) | ☐ | ☐ |
| **Level 2** — Area outside your home (porch, deck, driveway, yard) | ☐ | ☐ |
| **Level 3** — Places in your neighborhood | ☐ | ☐ |
| **Level 4** — Locations elsewhere in your town | ☐ | ☐ |
| **Level 5** — Locations outside your town | ☐ | ☐ |

**Scoring:**

- Assign 1 point for each “Yes” response from Levels 1–5 (Level 0 = 0 points if “Yes”).
- Total Simplified LSA Score = 0–5.
- Categorization:
  - Small life-space: ≤2
  - Medium life-space: 3–4
  - Large life-space: 5
