# Supplementary material for: Life-Space Mobility in Heart Failure With Preserved Ejection Fraction
Source: J Card Fail Intersect. Author manuscript; Available in PMC 2026 Jul 29. (PMC13410940; doi:10.1016/j.yjcafi.2025.12.013)
Supplement: 2 [file NIHMS2179308-supplement-2.pptx]

## Slide 1
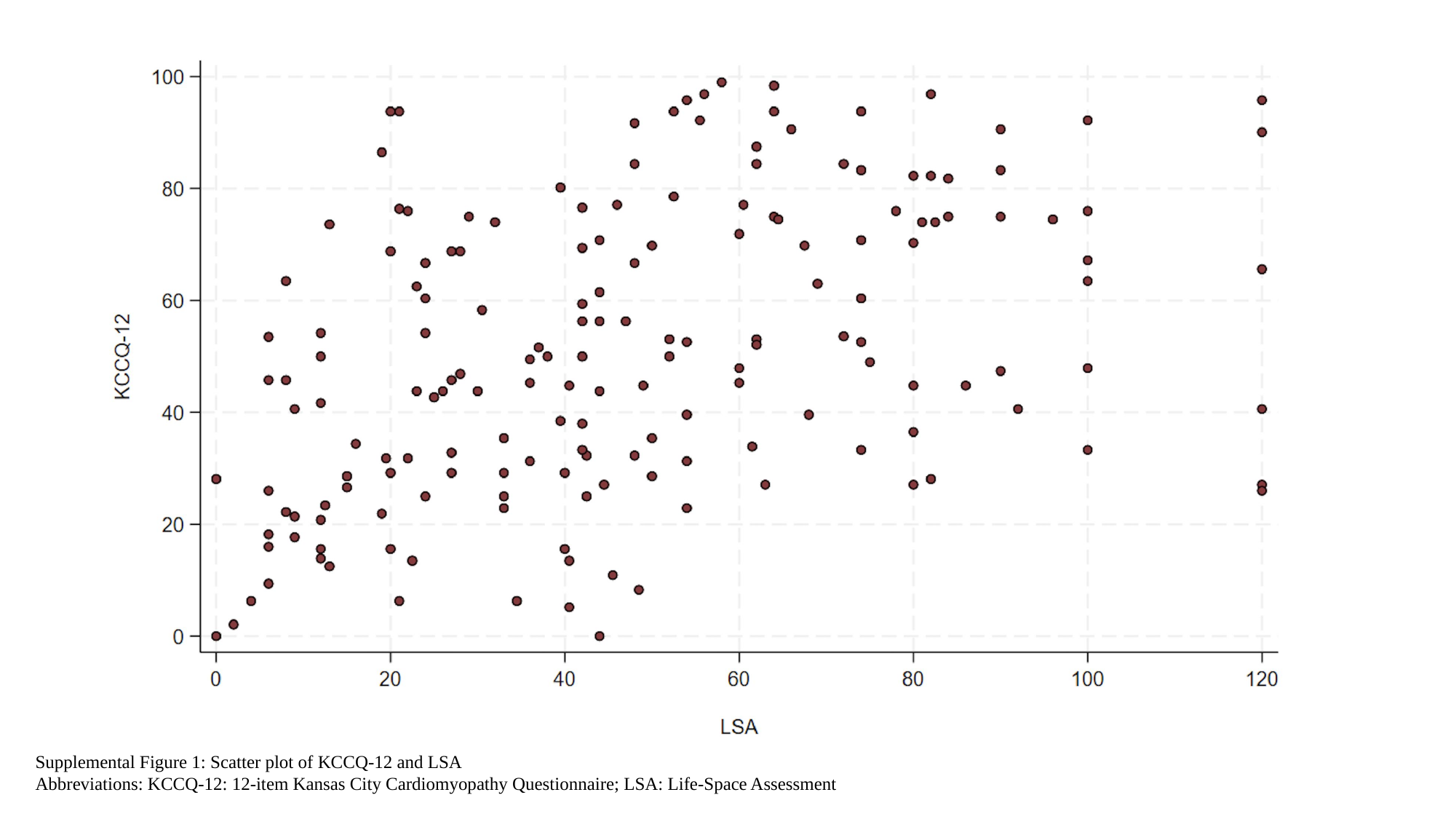

Supplemental Figure 1: Scatter plot of KCCQ-12 and LSA
Abbreviations: KCCQ-12: 12-item Kansas City Cardiomyopathy Questionnaire; LSA: Life-Space Assessment

## Slide 2
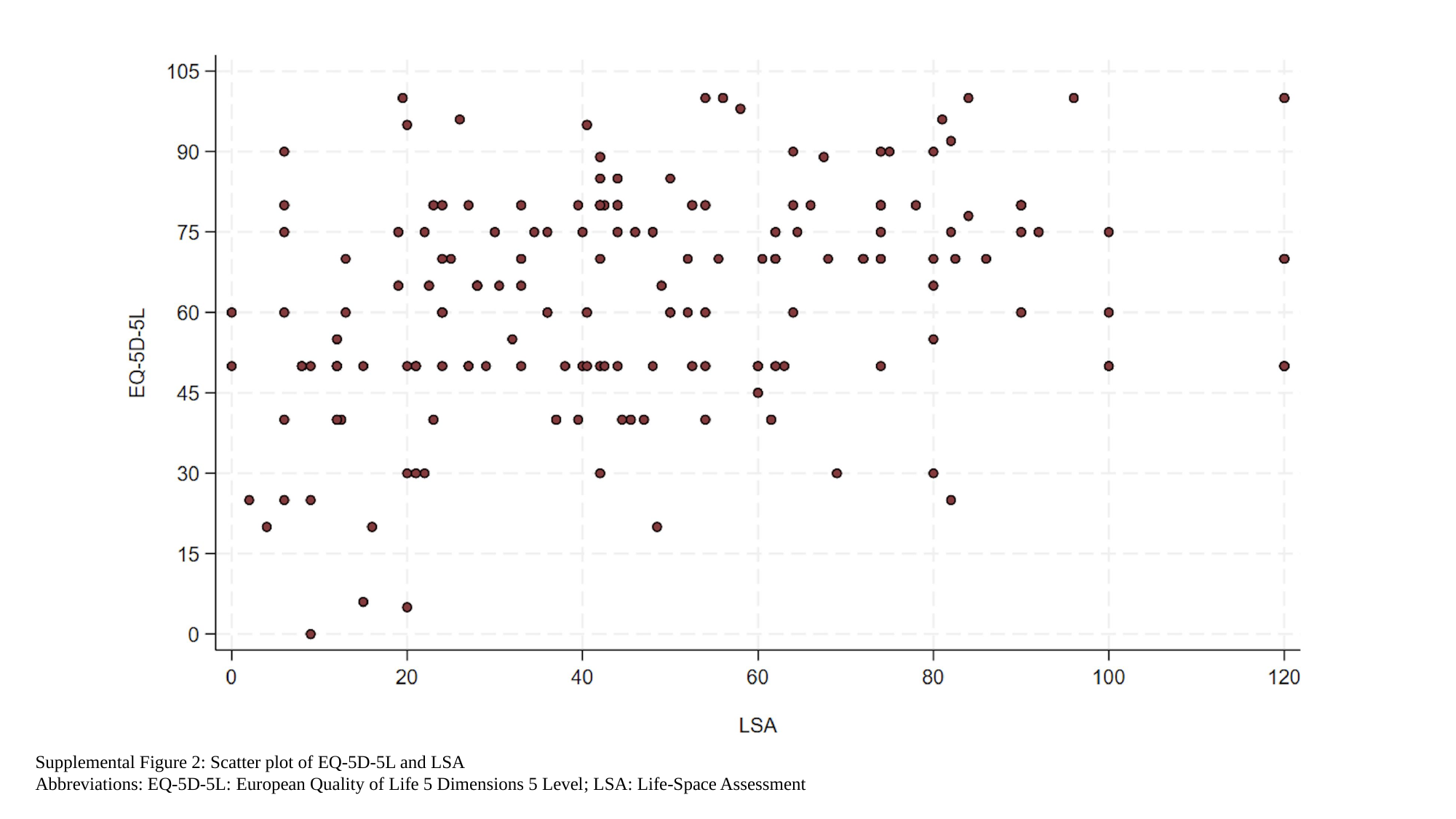

Supplemental Figure 2: Scatter plot of EQ-5D-5L and LSA
Abbreviations: EQ-5D-5L: European Quality of Life 5 Dimensions 5 Level; LSA: Life-Space Assessment

## Slide 3
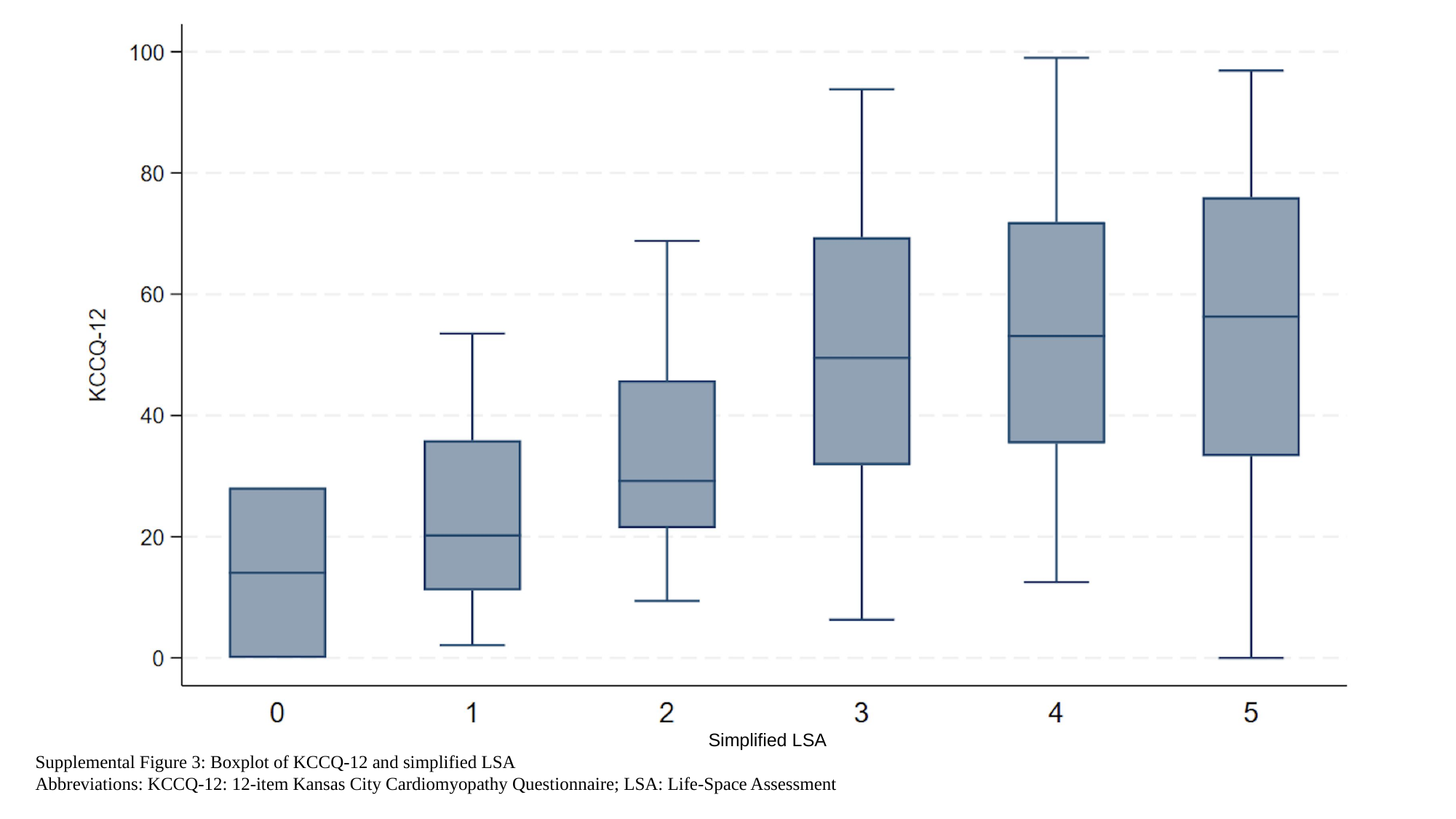

Simplified LSA
Supplemental Figure 3: Boxplot of KCCQ-12 and simplified LSA
Abbreviations: KCCQ-12: 12-item Kansas City Cardiomyopathy Questionnaire; LSA: Life-Space Assessment

## Slide 4
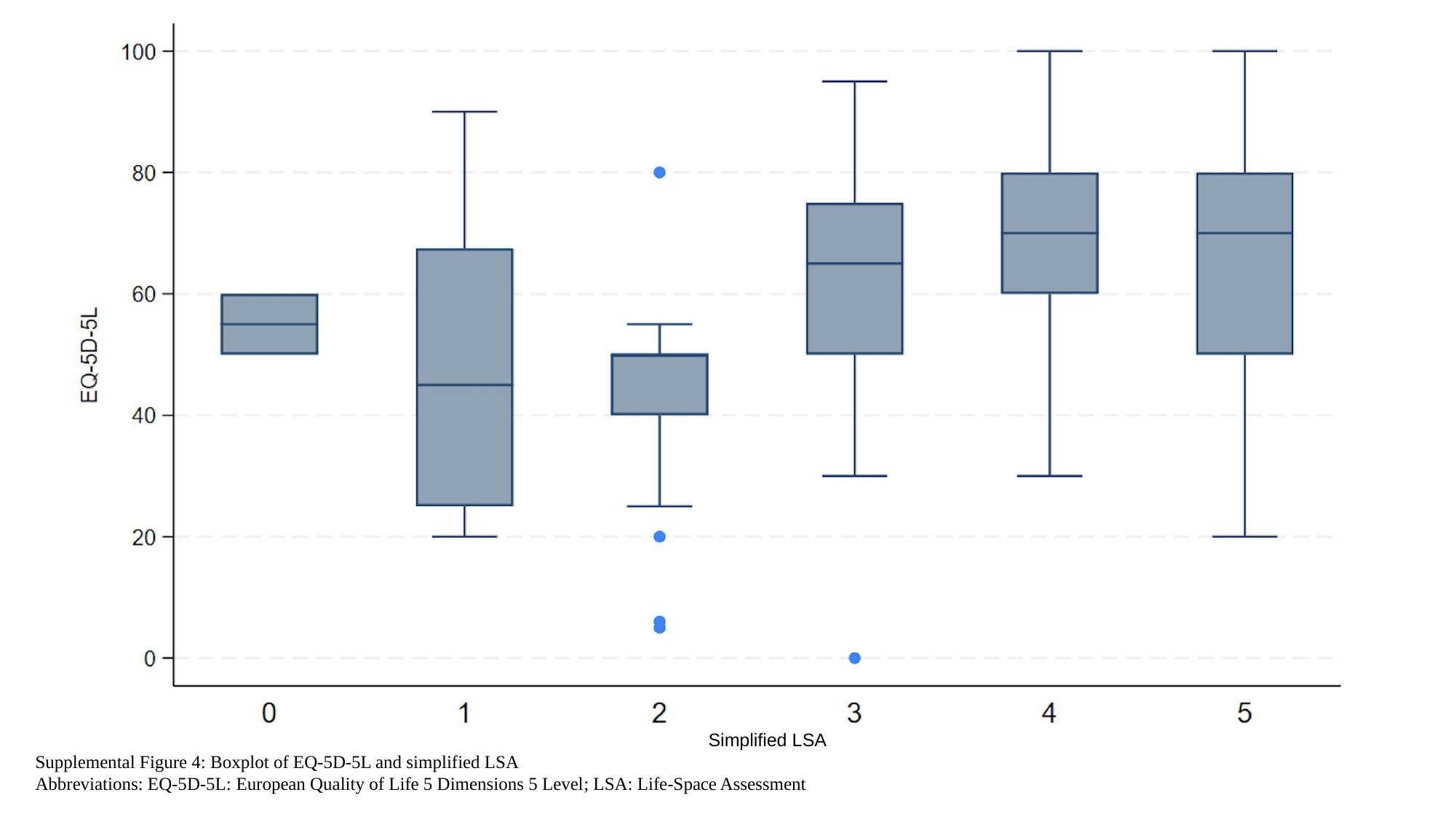

Simplified LSA
Supplemental Figure 4: Boxplot of EQ-5D-5L and simplified LSA
Abbreviations: EQ-5D-5L: European Quality of Life 5 Dimensions 5 Level; LSA: Life-Space Assessment

## Slide 5
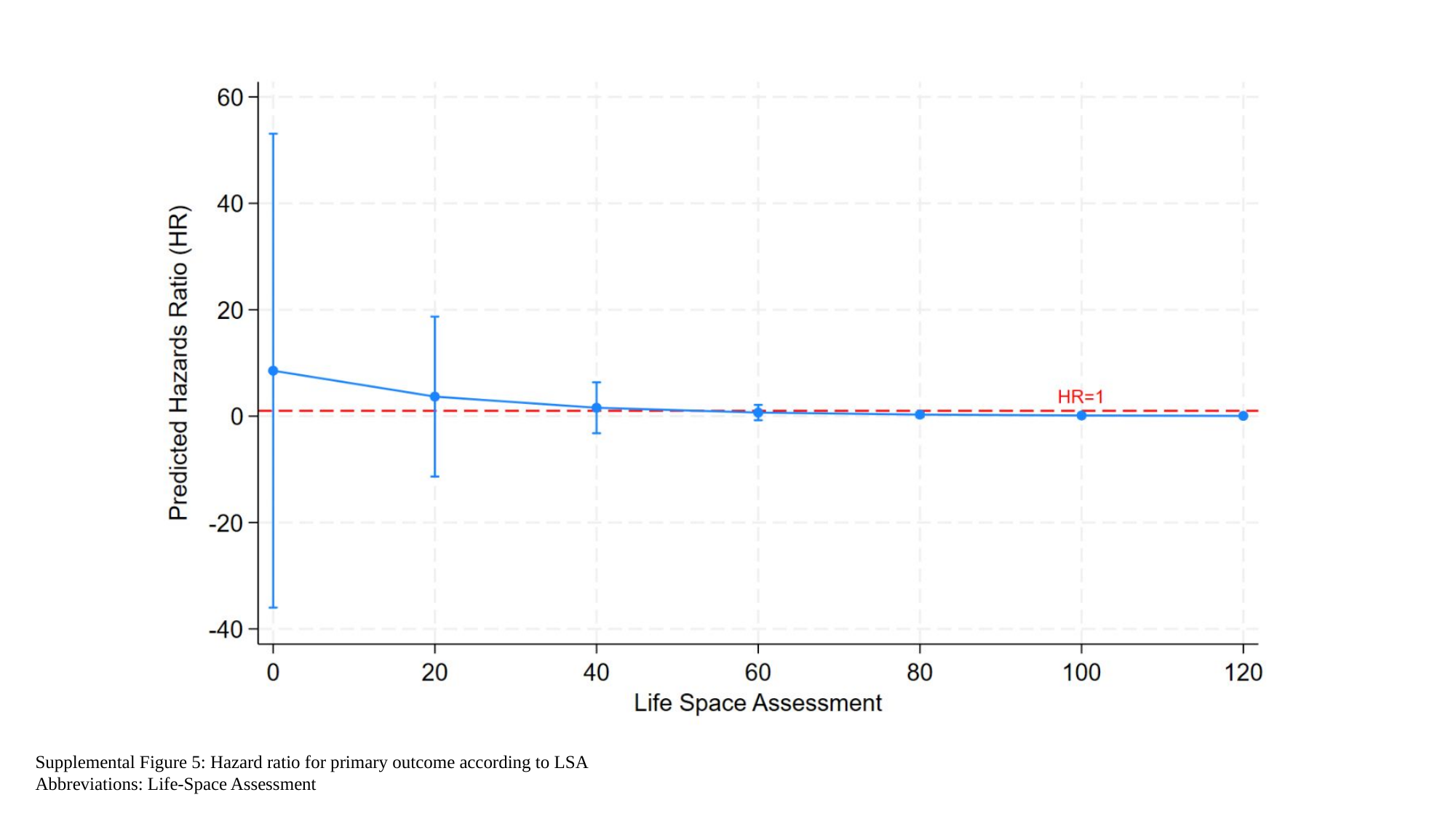

Supplemental Figure 5: Hazard ratio for primary outcome according to LSA
Abbreviations: Life-Space Assessment

## Slide 6
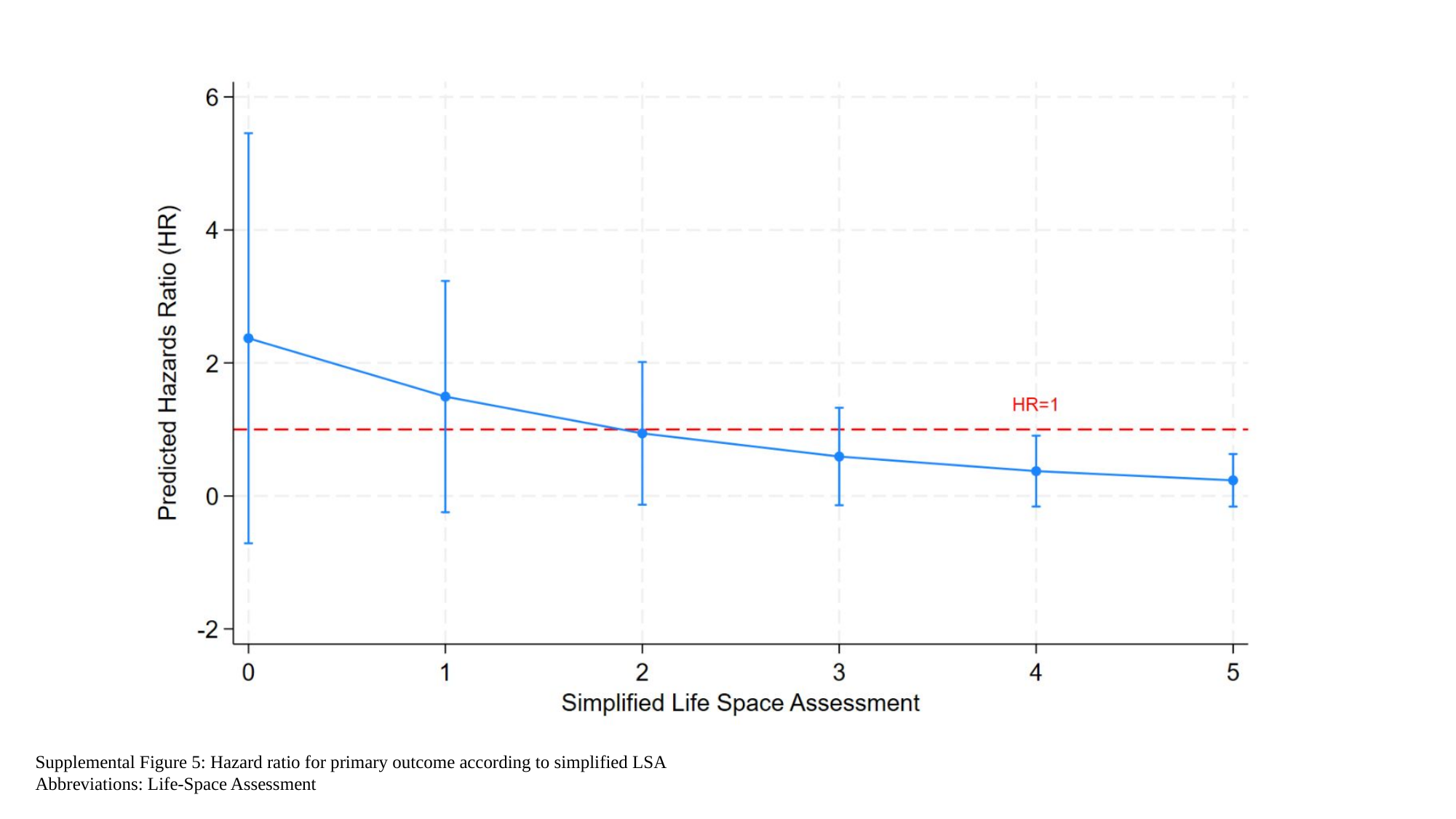

Supplemental Figure 5: Hazard ratio for primary outcome according to simplified LSA
Abbreviations: Life-Space Assessment
